# Decolonization of Global Health Law: Lessons from International Environmental Law

**DOI:** 10.1017/jme.2023.78

**Published:** 2023

**Authors:** Alexandra L. Phelan, Matiangai Sirleaf

**Affiliations:** 1: JOHNS HOPKINS UNIVERSITY, BALTIMORE, MD, USA; 2: UNIVERSITY OF MARYLAND, BALTIMORE, MD, USA

**Keywords:** International Law, Decolonization, Global Health, Climate Change, Equity

## Abstract

Global health law for pandemics currently lacks legal obligations to ensure distributional and reparative justice. In contrast, international environmental law contains several novel international legal mechanisms aimed at addressing the effects of colonialism and global injustices that arise from the disproportionate contributions to — and impacts of — climate change and biodiversity loss.

Global health law for pandemics currently lacks legal obligations to ensure distributional and reparative justice in critical areas, such as equitable access to vaccines, diagnostics, and therapeutics, and compensatory financing to strengthen health systems. This reflects the colonial history and present of international law for infectious diseases. In contrast, Global South efforts in the realm of international environmental law have propelled the development of novel international legal mechanisms to address global injustices arising from the disproportionate contributions to — and impacts of — climate change and biodiversity loss. These mechanisms seek to address international inequity, colonialism, and injustice, and include operational provisions to realize common but differentiated responsibilities (CBDR), access and benefit-sharing (ABS), and loss and damage (L&D). Early efforts to draft an international treaty for pandemic preparedness and response to build a more robust global health architecture provide new possibilities for decolonizing global health law. This is especially true when it comes to recognition of CBDR and ABS in global health law-making; however, caution is warranted when applying L&D to pandemic preparedness and response, as this framework could undermine global solidarity when it is needed most. This column explores the appropriateness of these international environmental law mechanisms, encouraging countries to consider integrating them in global health law reform processes.

## Embracing Common but Differentiated Responsibilities

CBDR is a principle associated with international environmental law that reflects the effort to achieve equity between richer countries in the Global North and poorer states in the Global South. Under this principle, richer countries agree to take on more stringent obligations to combat things like environmental concerns to reflect consumption and production patterns, as well as the unequal distributions of risks that result in more devastating environmental consequences for poorer countries.^1^ For example, CBDR is reflected in many aspects of the United Nations Framework Convention on Climate Change, including the differentiation between developing and developed countries in the Annex, as well as provisions in the 2015 Paris Agreement that stress the importance of financing and technology transfer for developing nations.^2^

CBDR is based in part on the principle of solidarity.^3^ It reflects the role of the Global South in shaping international law by demanding more equitable rules aimed at promoting substantive equality between developing and developed States, rather than mere formal equality. CBDR has two main elements: (1) common responsibility describes the shared obligations of two or more states towards the protection of a particular resource;^4^ and (2) differentiated responsibility describes a range of different burden-sharing arrangements that consider each nations’ particular circumstances, especially its ability to prevent, reduce and control the problem.^5^ Yet, this equitable approach to CBDR has been criticized as divisive, as countries located in the Global North tend to oppose having more demanding obligations. Some also express caution that adopting a principle primarily associated with international environmental law would have limited applicability in global public health law.

Early efforts to draft an international treaty for pandemic preparedness and response to build a more robust global health architecture provide new possibilities for decolonizing global health law. This is especially true when it comes to recognition of CBDR and ABS in global health law-making; however, caution is warranted when applying L&D to pandemic preparedness and response, as this framework can undermine global solidarity when it is needed most. This column explores the appropriateness of these international environmental law mechanisms, encouraging countries to consider integrating them in global health law reform processes.

Notwithstanding these concerns, CBDR remains a viable principle for adoption in global public health.^6^ The CBDR framework is not all or nothing — it provides an equitable and effective method for addressing mutual risks posed by a pandemic through differentiated and common obligations in ways that account for structural realities. The WHO’s latest draft of the pandemic treaty, released in June 2023, by the treaty Bureau, provides three options for States to consider. The first embraces the principle of CBDR in developing capabilities in pandemic prevention, preparedness, response, and recovery of health systems.^7^ The Bureau draft clarifies that “Parties that hold more capacities and resources relevant to pandemics should bear a commensurate degree of differentiated responsibility.”^8^ The second option does not differentiate responsibilities, but recognizes different capabilities, and that “unequal development in different countries… is a common danger.”^9^ Worryingly, this draft includes the option of not including CBDR as a principle in the treaty. However, if included and operationalized, CBDR could help address structural inequities in global health in ways that other frameworks do not.^10^

## Access and Benefit-Sharing

For the last two decades, countries in the Global South have sought to decolonize global health through global health law reforms that incorporate principles of ABS. This is because the use of genetic resources, including material of animal, plant, and microbial origin, to develop products, including medicines, is intrinsically linked to colonial acts of exploitation and extraction that inequitably benefit wealthy nations. Sovereign control over the use of genetic resources and the equitable distribution of their economic benefits has therefore been a core part of decolonization movements,^11^ such as the New International Economic Order and more recently through the lens of international biodiversity law. Under the Nagoya Protocol to the Convention on Biological Diversity, States developed an “access and benefit sharing” mechanism, establishing legal procedures for accessing genetic resources and the equitable sharing of the benefits that arise from the use of those resources.^12^ States have further developed several additional binding and non-binding legal instruments that implement access and benefit sharing regimes, including the Seed Treaty and the Pandemic Influenza Preparedness Framework, or modified benefits-sharing regimes reflecting the principles of ABS for genetic resources in areas beyond national jurisdiction, such as the recently adopted High Seas Treaty.

Under Article 12 of the pandemic treaty Bureau draft, Parties agree to establish an ABS system, with two options. The first includes the sharing of “biological materials with epidemic and pandemic potential” and associated sequence data, as well as pandemic-related products and other benefits, with details to be set by the Conference of Parties mechanism.The second option is for a “Pathogen Access and Benefit Sharing (PABS) System.” The goal of the PABS System is to establish a multilateral, fair, equitable, and timely system for sharing all pathogens with pandemic potential (not yet defined) and their genomic sequences, along with the benefits arising from their use. The provisions propose benefits-sharing at a minimum of 20% of real time access of pandemic-related products to WHO, distributed on a public health risk and needs basis or to treaty Parties, and prioritized to developing countries. However, this commitment alone would not prevent a repeat of the inequitable distribution of vaccines seen during the COVID-19 pandemic.^13^ The draft text therefore includes sharing benefits through collaboration with developing country manufacturers and WHO initiatives for technology transfer and capacity building. Alternative benefits-sharing provisions include an obligation on all Parties to include donations of pandemic-related products in government-funded purchase agreements, or, the weakest option, if a pandemic is declared, Parties that are able to agree to “make all possible efforts” to donate to countries in need. Some Global North countries and scholars criticize ABS as transactionalizing access to pathogens and the sharing of benefits.^14^ This risks dismissing the practical linkage between genetic resources, sequence data, and the development of vaccines, diagnostics, and therapeutics, as well as the decolonizing rationale underpinning the mechanism. However, even in light of that critique, establishing a multilateral mechanism like the PABS System is an implementable solution that is consistent with the objectives of ABS, while decoupling bilateral exchange of accessing pathogens, sequence data, and benefits. This has the effect of preserving their equitable connection while also facilitating the development of mechanisms for both accessing pathogens and sequence data, and for the global equitable distribution of vaccines, diagnostics, and therapeutics. This is notwithstanding the need for more robust reforms to build global manufacturing capacity, including technology transfer and the removal of intellectual property barriers.

## Loss and Damage

Not all legal mechanisms crucial for addressing global injustice in the environmental realm are directly transferrable to global health law and may inadvertently entrench inequities when applied to global health if not appropriately nuanced. Despite international efforts to reduce greenhouse gas emissions, the earth has already warmed 1.1ºC.^15^ Mitigating further warming and adapting to a changing climate are critical, but such steps will be insufficient to address the scale of impacts caused by climate change and it associated impacts on global health. There are already unavoidable losses and damages from the consequences of climate change, including acute events like extreme weather as well as slow-onset changes like sea level rises and desertification. These impacts are disproportionately borne by countries in the Global South: countries already particularly vulnerable to climate change despite contributing least to its cause. L&D is therefore an issue of climate justice. At COP27 in 2022, countries agreed to establish a dedicated fund for addressing L&D. This was a landmark moment in establishing commitments for L&D; however, the details of the fund’s governance, eligibility, and contributions have yet to be determined. Ideally, the fund should operate as a form of reparative justice, providing funding for economic and non-economic losses that would be financed by high income countries that have disproportionately contributed to climate change.

As a matter of both distributional and reparative justice, the pandemic treaty must similarly incorporate a financing mechanism that recognizes historical and ongoing injustices that determine which countries have the financial and technical resources to prevent, prepare for, and respond to pandemic threats. Yet despite this imperative for justice, such a mechanism is most appropriately addressed under the scope of CBDR, not L&D. Firstly, even climate risks in global health are avoidable through mechanisms for equitable access to adaptation measures such as vaccines, diagnostics, and therapeutics, rather than unavoidable L&D. Secondly, there is a pernicious risk of applying L&D to pandemics, assigning blame for the emergence of infectious diseases in resource constrained settings, rather than allocating responsibility derived from colonialism. This risks entrenching stigma and discrimination toward outbreak locations, compounding injustice while undermining, rather than building, the necessary international solidarity for global public health. Instead, appropriately applying L&D to pandemics would involve reparatory justice for the losses and damage experienced in already resource-constrained settings arising out of the Global North’s actions, such as engaging in acts of vaccine apartheid. An L&D framework could also be aptly utilized to account for inactions by countries in the Global North, such as failing to finance and support the building of robust public health system capacities in countries constrained from doing so as a result of colonialism and neo-colonialism.

## Conclusion

Global health law reforms can help foster the progressive development of international law in ways that can contribute to decolonizing global health. However, if global health law is to achieve its normative goal of equity, and facilitate the international solidarity long called for and especially needed during the COVID-19 pandemic, it must embrace progressive legal mechanisms for global health with justice. CBDR and ABS are legal mechanisms from international environmental law that have the potential to be integrated into global health law, while L&D may not be tailored for global public health but could be more narrowly adapted. Fundamentally, it remains to be seen whether ongoing reforms will be truly transformational and assist with the much-needed decolonization of global health law.

## Note

The authors have no conflicts to disclose.
